# Human in silico trials for parametric computational fluid dynamics investigation of cerebrospinal fluid drug delivery: impact of injection location, injection protocol, and physiology

**DOI:** 10.1186/s12987-022-00304-4

**Published:** 2022-01-28

**Authors:** Mohammadreza Khani, Goutham Kumar Reddy Burla, Lucas R. Sass, Ostin N. Arters, Tao Xing, Haiming Wu, Bryn A. Martin

**Affiliations:** 1 Alcyone Therapeutics, Lowell, MA USA; 2 Department of Chemical and Biological Engineering, The University of Idaho, Moscow, ID USA; 3 Department of Mechanical Engineering, The University of Idaho, Moscow, ID USA; 4 Biogen, Cambridge, MA USA

**Keywords:** Intrathecal drug delivery, Ventricular drug delivery, Cisterna magna drug delivery, Cerebrospinal fluid, Computational fluid dynamics, In vitro model, Central nervous system, Multiphase solute transport, Magnetic resonance imaging, Biofluid mechanics, Biomechanics

## Abstract

**Background:**

Intrathecal drug delivery has a significant role in pain management and central nervous system (CNS) disease therapeutics. A fluid-physics based tool to assist clinicians in choosing specific drug doses to the spine or brain may help improve treatment schedules.

**Methods:**

This study applied computational fluid dynamics (CFD) and in vitro model verification to assess intrathecal drug delivery in an anatomically idealized model of the human CSF system with key anatomic features of the CNS. Key parameters analyzed included the role of (a) injection location including lumbar puncture (LP), cisterna magna (CM) and intracerebroventricular (ICV), (b) LP injection rate, injection volume, and flush volume, (c) physiologic factors including cardiac-induced and deep respiration-induced CSF stroke volume increase. Simulations were conducted for 3-h post-injection and used to quantify spatial–temporal tracer concentration, regional area under the curve (AUC), time to maximum concentration (T_max_), and maximum concentration (C_max_), for each case.

**Results:**

CM and ICV increased AUC to brain regions by ~ 2 logs compared to all other simulations. A 3X increase in bolus volume and addition of a 5 mL flush both increased intracranial AUC to the brain up to 2X compared to a baseline 5 mL LP injection. In contrast, a 5X increase in bolus rate (25 mL/min) did not improve tracer exposure to the brain. An increase in cardiac and respiratory CSF movement improved tracer spread to the brain, basal cistern, and cerebellum up to ~ 2 logs compared to the baseline LP injection.

**Conclusion:**

The computational modeling approach provides ability to conduct in silico trials representative of CSF injection protocols. Taken together, the findings indicate a strong potential for delivery protocols to be optimized to reach a target region(s) of the spine and/or brain with a needed therapeutic dose. Parametric modification of bolus rate/volume and flush volume was found to have impact on tracer distribution; albeit to a smaller degree than injection location, with CM and ICV injections resulting in greater therapeutic dose to brain regions compared to LP. CSF stroke volume and frequency both played an important role and may potentially have a greater impact than the modest changes in LP injection protocols analyzed such as bolus rate, volume, and flush.

**Supplementary Information:**

The online version contains supplementary material available at 10.1186/s12987-022-00304-4.

## Background

Cerebrospinal fluid (CSF) has a complex and dynamic movement that allows solutes to disperse around the brain and within the ventricles. CSF has a viscosity and density similar to water at body temperature and resides in the subarachnoid space (SAS) and ventricles of the brain with a total volume ranging from 250 to 400 mL in healthy adult humans [1, 2]. CSF moves in an oscillatory manner with velocities ranging from 0 to 15 cm/s [3] in response to periodic cerebral blood volume variations during the cardiac and respiratory cycle [4, 5]. Injection of tracers into the CSF and computational modeling has shown that slow “steady-streaming” CSF velocities, on the order of ~ 0.1 mm/s, lead to net movement of tracers throughout the CSF system [6]. This transport can be observed by cintigraphic imaging [7], positron emission tomography [8], and contrast enhanced magnetic resonance imaging using gadolinium [9]. Movement of CSF tracers into the central nervous system tissue along paravascular spaces has been described [10]. The exact mechanism(s) by which this transport takes place has been hypothesized to be due to fluid advection, various types of diffusion, and/or fluid structure interaction [11].

**Fig. 1:**
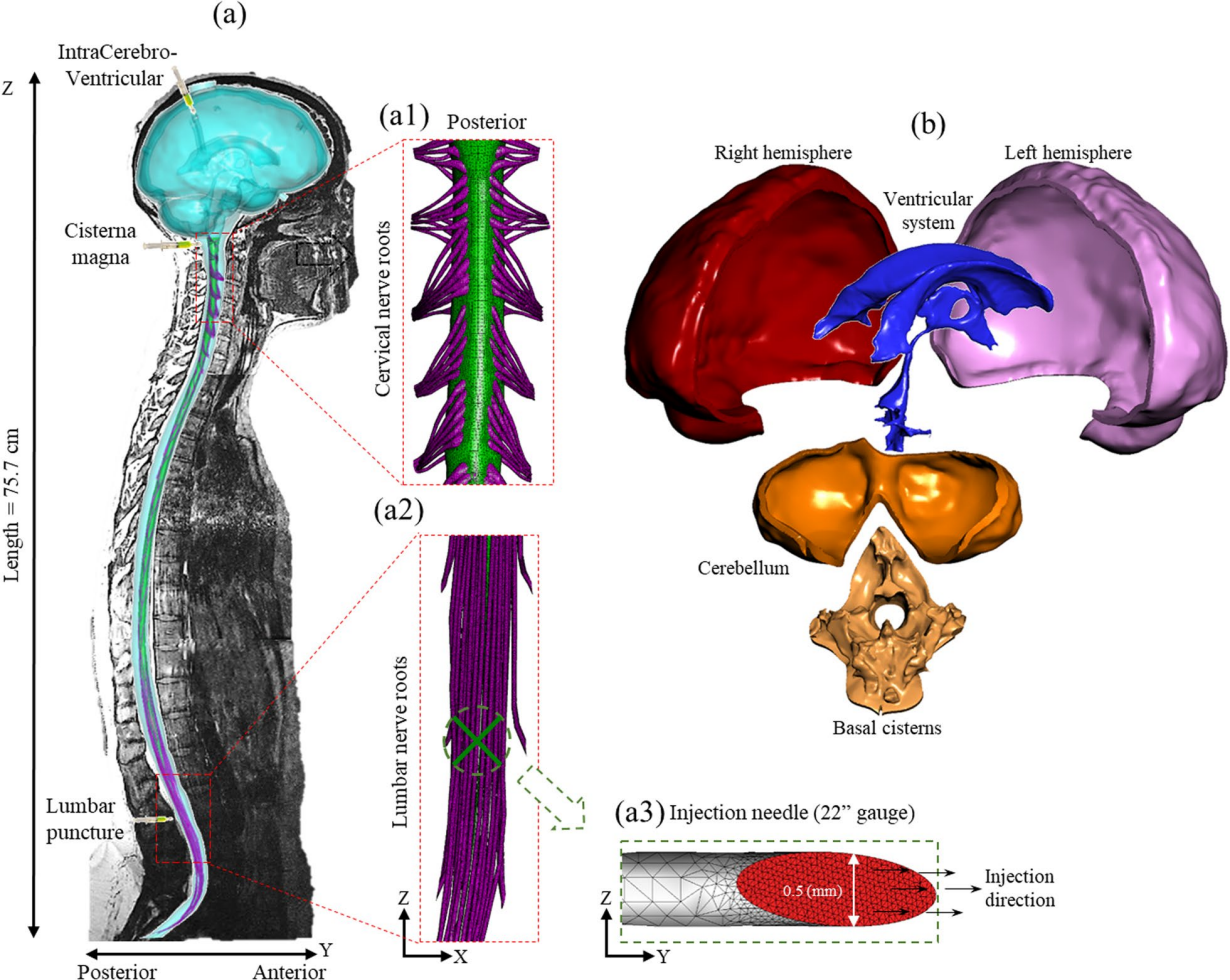
Overview of numerical model based on subject specific MRI measurements. **a** Computational model of the entire CSF space for the human analyzed superimposed on a T2-weighted MR image used to acquire subject-specific anatomy and natural CSF oscillation. (**a1**) Magnification of cervical nerve roots (**a2**) Magnification of lumbar nerve roots (**a3**) Visualization of the needle surface mesh used for tracer injection. **b** Cranial SAS compartments consisting of ventricular system, cerebellum, basal cistern, and cortical subarachnoid space (left and right)

The combination of close proximity and naturally occurring solute transport along the neuro-axis make CSF an intriguing pathway for delivery and removal of solutes to and from the central nervous system (CNS). One advantage of bypassing the blood–brain-barrier through CSF drug delivery is potential for reduction of systemic toxicity because of increased dose efficiency to the CNS [12–14]. CSF drug delivery involves solute injection from a catheter or needle that is inserted at various locations within the CSF system including a) lumbar puncture (LP) [15], b) intracerebroventricular injection (ICV), c) cisterna magna (CM) [16], d) cortical subarachnoid space [17], and combinations thereof (Fig. 1). Pain and spasticity management with Baclofen can be applied via catheter insertion in the lumbar intrathecal space [18] or cervical spine [19]. CM drug injection has been applied for ongoing gene therapy trials [20, 21]. ICV is widely used in treating diseased mice models by introducing pharmaceuticals, therapeutic RNAs, plasmid DNAs, and viral vectors [10]. Ventricular treatment can also be used in human patients for neurodegenerative disorders such as spinal muscular atrophy or supplement chemotherapy in gliomas with neurotrophic factors [11].

While CSF drug delivery is of growing interest, we have relatively little knowledge about the factors impacting CSF drug dispersion. These factors include: (1) catheter placement location, (2) drug injection protocol including flow rate, bolus volume and flush volume, (3) CSF physiology including cardiac-related stroke volume and respiratory fluctuations, (4) patient disease-specific anatomy, (5) drug injection formulation, baricity and chemical properties. After dispersion of a drug in the CSF, it is absorbed into the CNS tissue and other outflow pathways. This is also a complex process that involves movement of solutes across the pia-arachnoid complex membranes, along peri- and/or paravascular spaces, and into the interstitial spaces and ultimately into neurons. Understanding these factors is needed to minimize therapy risk, complications, and costs while maximizing benefits [13, 14]. In particular, some CSF-delivered gene therapy drugs cost as much as US$1 million per patient [22] and other CSF-delivered medications such as Nusinersen can involve multiple expensive injections over a period of years [23].

Computational fluid dynamics (CFD) modeling is a powerful tool for conducting in silico trials that can be applied to help understand the above-mentioned factors impacting CSF drug dispersion. Several numerical modeling studies have investigated intrathecal drug delivery solute transport (Table 1). Kuttler et al. [24] developed and implemented a simplified spinal SAS geometry to model CSF transport. Their results provided some primary insights about drug distribution in CSF with LP injection. Tangen et al. [25] detected mixing effects due to microanatomical structures in a subject-specific SAS model that enhanced drug dispersion. Considering physiological principles, Hsu et al. assessed the influence of CSF oscillations on intrathecal drug delivery based on 2D geometry from anatomical images. Myers group investigating intrathecal drug delivery with an idealized 3D elliptical geometry to assess potential effects of flow rate and catheter size and orientation [26]. Drug transport in the nervous tissue was numerically investigated as well, both at the spinal and cerebral level [27]. The effect of catheter position and angle was investigated by Pizzichelli et al. [28] and Haga et al. [29] within the cervical subarachnoid space. Complementary experiments on non-human primates were also performed to investigate tissue penetration mechanisms [16, 30].

**Table 1:** Summary of previous in silico and in vivo CSF solute transport studies conducted in the literature to investigate the impact of physiology, injection protocol, and injection location (↓: indicates a decrease in tracer transport to the brain, ↑: increased, ↔ : not changed, ✓: indicates one simulation performed)

Author et al. (year)		Physiology			Injection protocol			Injection location		
		Heart rate	Stroke volume	Respiration	Bolus volume	Bolus rate	Flush	LP	CM	ICV
CFD	Khani (current)		**↑**	**↑**	**↑**	**↓**	**↑**	**✓**	**↑**	**↑**
	Tangen (2017) [47]	**↑**			**↑**		**↑**	**✓**		
	Kuttler (2010) [24]					**↓**		**✓**		
	Haga (2017) [36]					**↑**		**✓**		
	Hsu (2012) [48]	**↑**	**↑**					**✓**		
	Myers (1996) [26]					**↓**		**✓**		
In vivo	Watts (2019) [38]							**✓**		
	Verma (2020) [8]			**↑**	**↑**			**✓**		
	Ringstad (2018) [49]							**✓**		
	Whiteside (2001) [50]							**✓**		
	Malinovsky (1999) [51]				**↑**			**✓**		
	Sullivan (2020) [52]				**↑**			**✓**		
	Ringstad (2018) [49]							**✓**		
	Wolf (2016) [40]				**↑**		**↑**	**✓**	**↑**	
	Meyer (2015) [41]							**✓**		**↑**

*LP* Lumbar Puncture, *CM* Cisterna Magna, *ICV* IntraCerebroVentricular

Despite the outstanding contributions available in literature, to the best of our knowledge, a 3D anatomically detailed model of the entire CSF system has not been used to investigate the possibility and a range of effects of injection parameters, injection locations, and CSF physiology on CSF drug delivery. The present study presents a numerical model of drug injection based on a patient-specific 3D geometry of the entire SAS with idealized anatomical structures.

## Methods

Our overall approach was to build a subject-specific multiphase CFD simulation to investigate the impact of the following parameters on tracer distribution within the CSF: (a) catheter position, (b) injection parameters (bolus volume, bolus rate and CSF flush) and (c) physiological parameters (deep respiration and stroke volume) (Table 2). Each simulation was conducted for a period of three hours after tracer injection as our focus was on the short-term tracer pharmacokinetic transport within the CSF and not long-term tracer absorption into the CNS tissue. For comparison, tracer dosage was held constant across all simulations and quantified in terms of spatial temporal concentration throughout the CSF system. Numerical simulation results were checked for mesh independence and verified with a subject-specific in vitro bench top model as previously described in our research.

**Table 2:** Summary of injection parameters simulated

Description	Name	Location	Injection flow rate (mL/min)	Injection bolus volume (mL)	Flush volume (mL)	CSF Stroke Volume (mL)	Respiration
Lumbar Puncture (baseline)	LP	L3/4	5	5	0	1	NA
Cisterna Magna	CM	FM/C1	5	5	0	1	NA
IntraCerebroVentricular	ICV	FM/C1	5	5	0	1	NA
Lumbar puncture with 5X higher Bolus Rate	5X-Bolus rate	L3/4	25	5	0	1	NA
Lumbar puncture with 3X higher Bolus Volume	3X-Bolus volume	L3/4	5	15	0	1	NA
Lumbar puncture with 2X higher stroke volume	2X-Stroke volume	L3/4	5	5	0	2	NA
Lumbar puncture with 5 mL flush after injection	5 mL-Flush	L3/4	5	5	5	1	NA
Lumbar Puncture with deep respiration waveform super imposed on cardiac flow waveform	Deep respiration	L3/4	5	10	0	1	Yes

### Geometry

A subject-specific 23-year-old female CSF system geometry [31] including 31 pairs of anatomically idealized spinal cord nerve roots, filum terminale, thecal sac, and a detailed intracranial CSF space geometry with complete intraventricular cisterns was used to simulate the CSF flow dynamics (Fig. 1). The tracer was injected via a 22-gauge needle inserted at L3-L4 and FM-C1 vertebral level within the posterior SAS for the LP and CM injection location, respectively. Needle geometry was specified based on manufacturer specified needle dimensions with an internal diameter of 0.5 mm (J-#529H, 22GX3.5″ spinal needle, Jorvet). To simulate ICV drug delivery, tracer was injected at the posterior lateral ventricular horn.

An unstructured tetrahedral computational mesh was generated using ANSYS ICEM CFD (version 19.2, Canonsburg, PA). The computational mesh was further refined with a prism layer near boundaries and near the needle tip. To allow computation of CSF velocity profile at the needle termination, the mesh also included the internal needle lumen. The final computational mesh covering the needle, cranial, and spinal SAS, and ventricles had 18.7 million cells.

### Flow conditions

**Fig. 2:**
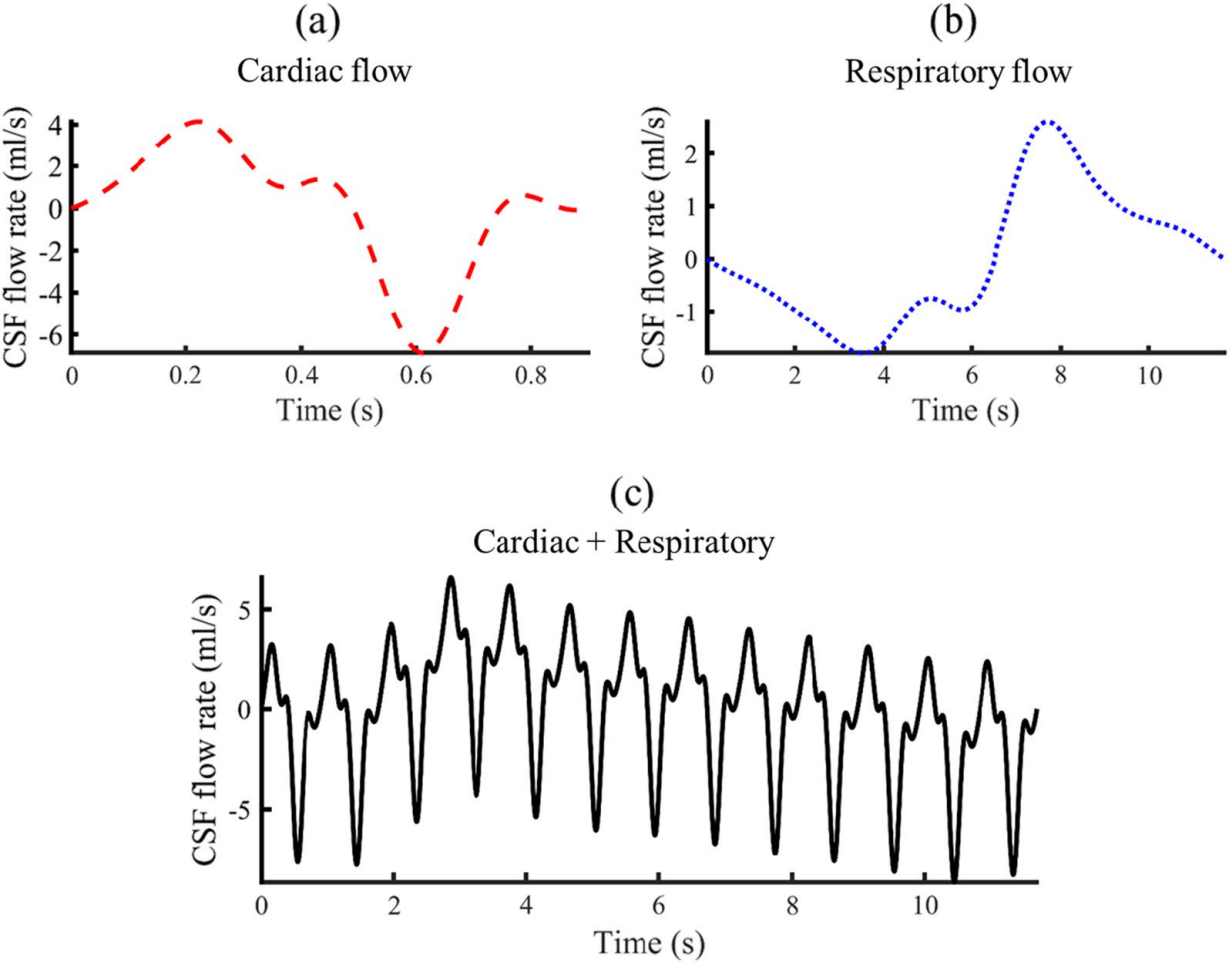
CSF flow waveforms measured by PCMRI at C2-3 vertebral location. Dash line indicates cardiac flow waveform. Dotted line indicates respiratory flow waveform by Yildiz et al. [4]. Sloid line indicates final flow waveform achieved by summation of respiratory and cardiac flow wave from

The model was oriented with the subject lying on the side to represent a typical patient position for LP injection. To represent CSF oscillations around the brain and spinal cord, an oscillatory velocity inlet boundary condition was defined at the caudal opening of the model by applying a user defined function. A representative CSF flow rate waveform was applied with a 1.0 mL stroke volume at the C2-C3 vertebral level (Fig. 2a). Ventricular CSF production rate was specified to be 0.4 mL/min or ~ 576 mL/day based on the literature [32]. A zero-pressure outlet boundary condition was defined at the cranial opening as an elimination route or absorption location. No-slip boundary conditions were imposed at the model walls (dural, pial and intraventricular spaces). The CSF was modeled as an incompressible fluid identical to water at room temperature (as the in vitro model experiments were conducted at room temperature) with a density of 998.3 kg/m^3^ and viscosity of 0.89 mpa-s.

Deep respiration impact was only considered in one simulation by adding the respiratory flow waveform described by Yildiz et al. [4] (Fig. 2b). First, the respiratory flow amplitude was scaled to be 40% of the cardiac flow. Second, the cardiac flow waveform was repeated 13 times to span the entire respiration cycle and then added to the respiratory flow. The final flow wave form was shifted to have zero net-flow through each respiratory and cycle (Fig. 2c).

ANSYS Fluent 19.2 (Canonsburg, PA) was used to solve the oscillatory CSF flow field using a laminar viscous model. The convergence criterion was set to 1E-06. A total computation time of two weeks was needed to simulate three hours of real-world time running in parallel mode with 126 GB RAM and 78 processors. Numerical sensitivity studies for time-step size and mesh resolution were carried out for axial distribution of cross-sectional average tracer concentration and tracer concentration across a rake of points located at the basal cistern, thoracic spine and lumbar spine at 3 h. The possible inaccuracy between two phases based on the numerical diffusion was addressed by refining the mesh near the injection location. A time-step size of 0.01 s and maximum mesh size of 0.5 mm was used for each simulation. The PISO Scheme (pressure-implicit with splitting of operators) with PRESTO! (pressure staggering options) pressure discretization method was used to solve the flow equations. Second-order upwind and first-order upwind was used for discretization of momentum and volume fraction. Under relaxation factors were set to default values. The implicit formulation was used for volume fraction parameters and a dispersed model was used for phases interface modeling. More details about the flow and phase equations is provided in our previous study [31].

### Injection and physiological parameters

A summary of injection and physiological parameters utilized for each simulation is indicated in Table 2. A “baseline” injection rate for the LP simulation was set to 5 mL/min and the bolus volume was 5 mL. Two more simulations were performed to study the effect of injection location. First by moving the needle to the CM, between the Foramen magnum and C1 vertebrae within the posterior SAS. The second simulation (ICV) was performed by injecting directly to the lateral ventricles through the left ventricle. The flow rate and bolus volume were specified to be identical to the LP simulation for both CM and ventricular injections.

Our focus was to analyze the impact of injection parameters and physiological changes only under LP injection scenario. The effect of flush (5 mL-Flush) was investigated by injecting 5 mL of water in 1 min immediately after the end of bolus injection. To study the effect of injection bolus volume (3X-Bolus volume), the injection rate considered to be constant (5 mL/min) and the injection bolus volume increased 3X (15 mL). To keep the tracer dosage constant, the injection volume fraction was set to 0.33 for this larger bolus. To study the effect of injection bolus rate (5X-Bolus rate), the bolus volume was considered to be constant (5 mL) and the injection rate was set to 25 mL/min.

Two more simulations were performed to study the effect of physiological parameters of stroke volume and respiration. The first simulation (2X-Stroke volume) was performed by increasing the CSF stroke volume, defined as the one half of the integrated flow for the entire CSF oscillation cycle, from 1 to 2 mL. This degree of change was selected to be representative of potential alterations in CSF stroke volume across subjects. For this simulation, only CSF flow magnitude was increased while cardiac cycle frequency was held constant. The second simulation studied the impact of deep respiration by adding the deep respiration flow waveform measured by Yildiz et al. [4] to the current cardiac flow waveform.

#### Multiphase simulation

Details about the multiphase simulation model is provided in our previous study [31]. In brief, tracer dispersion was modeled using an ANSYS mixture multi-phase model. Molecular diffusion of the tracer was not included in the model, as previously assumed to be negligible by Khani et al. [31]. To determine concentration profiles over 3-h after injection, we determined the solute transport due to the steady-streaming CSF velocity field. Steady-streaming velocities were determined based on the average of one CSF flow cycle with transient effects eliminated by discarding the first flow cycle for the calculation. Tracer distribution was quantified in terms of spatial–temporal cross-sectional average concentration and 3D tracer distribution contour at multiple time points of 5, 15, 30, 60 and 180 min. Pharmacokinetic parameters for tracer were presented as the maximum concentration (C_max_) and time at which the maximum concentration occurred (T_max_) for each axial location. Tracer exposure, or integrated value of tracer concentration over time (3 h in this study), is defined by the area under the curve (AUC).

### In vitro* experimental model*

**Fig. 3:**
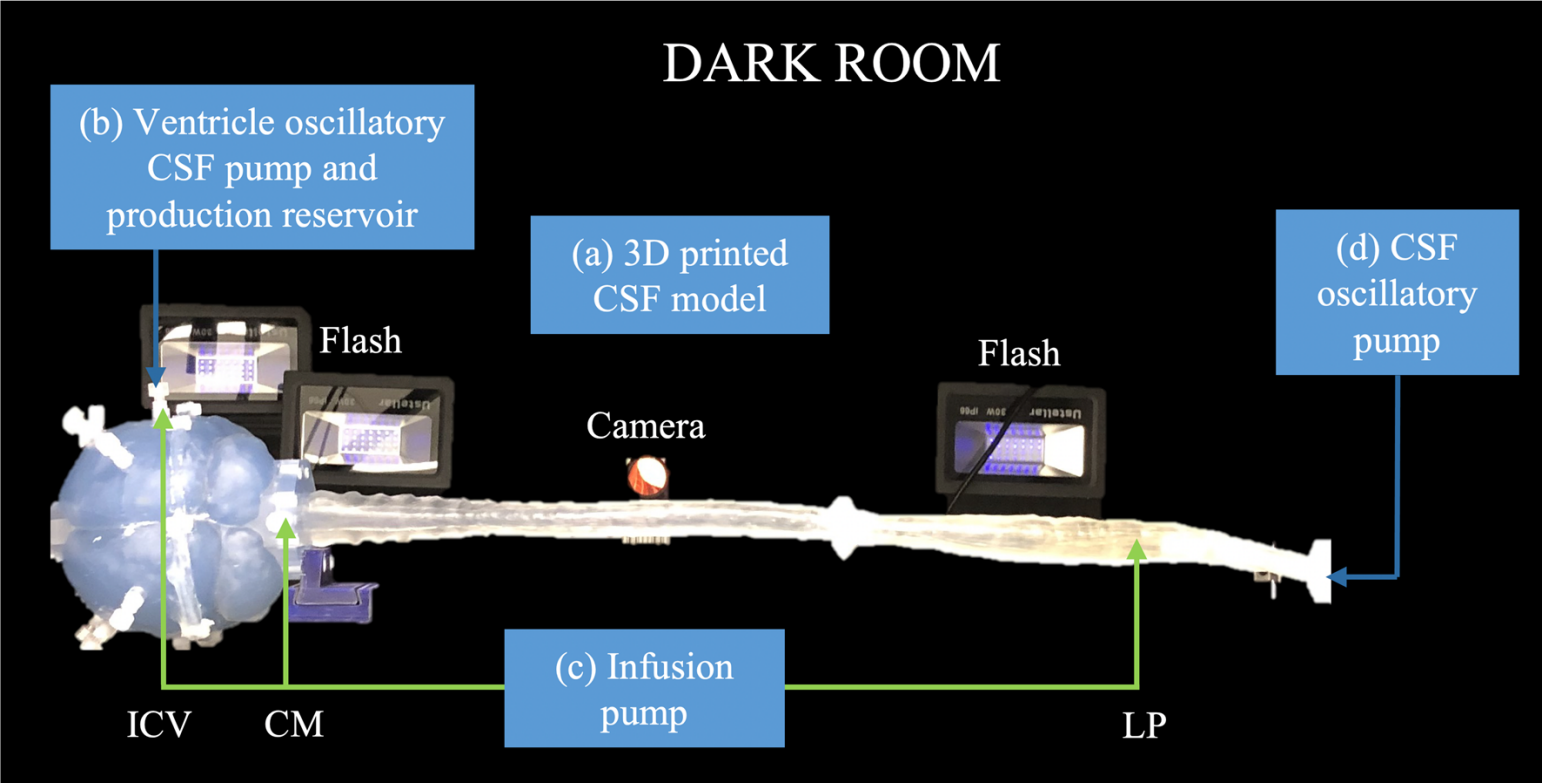
Overview of in vitro system and bench top experiment setup **a** 3D printed n-vitro model of the CSF system with placement of flash lightning and imaging camera. **b** CSF reservoir tank and custom ventricular oscillatory CSF flow pump in the ventricles. **c** Continuous injection pump for tracer injection at ICV, CM, and LP. **d** Oscillatory pump to induce CSF oscillations in the SAS to match the CSF flow field acquired by phase contrast-MRI

To help verify the numerical model results, an in vitro CSF model was constructed with an identical fluid domain geometry (Fig. 3). The shell was divided into cranial, upper thoracic, and lower spine pieces not exceeding the maximum build size of the 3D printer. Details about the in vitro model geometry is provided in our previous study [31]. In brief, to visualize drug distribution within the CSF, an aqueous solution of fluorescein sodium was used to mimic a small molecule drug. Experiments were conducted with deionized water since the nature of physical transport is based on oscillation and vorticity, which are largely independent of chemical composition of the bulk fluid [33]. The model was printed with Somos® WaterShed XC 11122. The model was flushed after each experiment, with no tracer absorption into the model noted.

A continuous flow syringe pump was used for CSF production originating from the ventricles. Pump flow rates were verified by bucket-and-stopwatch before each experiment. An optical imaging system was used to quantify spatial–temporal tracer concentration as previously described by Khani et al. [31]. Reliability of in vitro results was determined by conducting triplicate experiments of each case and quantifying the average magnitude difference of the mean experimental result for each experiment.

To calibrate the imaging system, a group of data sets were needed as a standard for the actual fluorescein concentration with respect to the luminescence captured in the digital images. Thus, the system was filled with a uniform fluid of multiple fluorescein concentrations and images were captured with each concentration. Collection of each calibration image-set was replicated three times to improve the robustness of the measurement. These image sets were then used to interpolate raw luminescent intensity values into known molar concentrations throughout the model.

### Verification of results with in vitro simulations

#### Linear regression of CFD versus in vitro and in vitro repeatability analysis

A detailed comparison of numerical and in vitro results was performed by the following correlation analysis similar to that previously conducted by our group [31]. The in vitro results were compared to the numerical simulations for each case in terms of the spatial–temporal difference of tracer concentration over 3-h. For each z-location and time, the numerical concentration was plotted against the corresponding value obtained from the in vitro model. A linear regression comparison along with a Bland–Altman analysis were subsequently performed.

## Results

### Impact of injection location

**Fig. 4:**
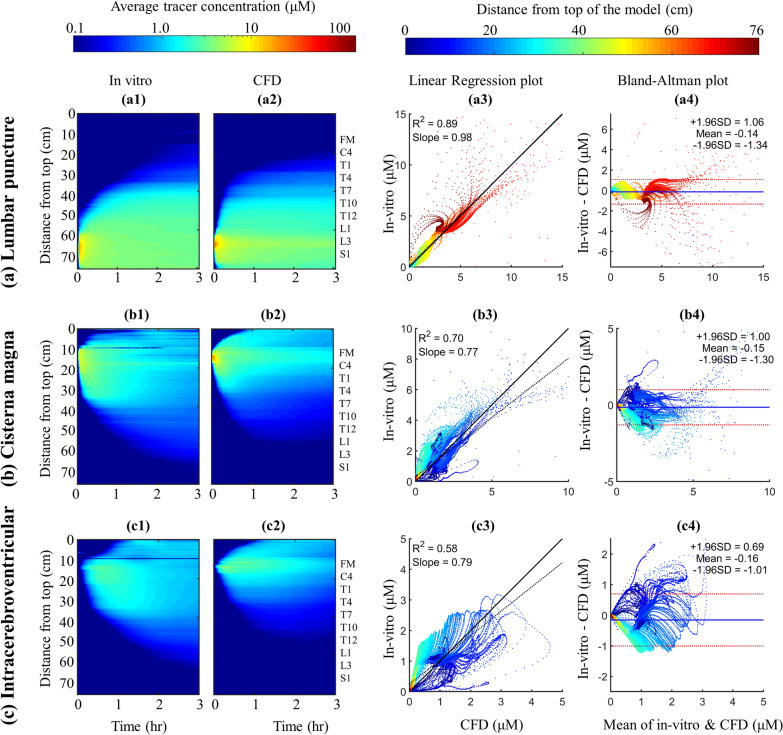
Cross-sectional average tracer concentration over 3-h and regression plots to compare numerical simulation with in vitro model at different injection locations **a** Lumbar puncture, **b** Cisterna magna and **c** Intracerebroventricular injection. **a1**, **b1**, **c1** Spatial temporal plot for cross-sectional average tracer concentration for CFD at LP, CM and ICV, respectively. **a2**, **b2**, **c2** Spatial temporal plot for cross-sectional average tracer concentration for in vitro model at LP, CM and ICV, respectively. **a3**, **b3**, **c3** the linear correlation plots for agreement of in vitro and numerical simulation results for spatial–temporal cross-sectional average tracer concentration over 3-h at LP, CM and ICV, respectively. The linear regression is shown in black dashed line and the idea line is shown in black solid line. **a4**, **b4**, **c4** Bland–Altman plots for agreement of in vitro and numerical simulation results for spatial–temporal cross-sectional average tracer concentration over 3-h at LP, CM and ICV, respectively. The limit of agreement (95% confidence intervals) lines are shown in dashed lines and the mean value of differences between CFD and in vitro is shown in solid blue line

In particular, Fig. 4 shows the tracer spatial temporal distribution for LP (Fig. 4a), CM (Fig. 4b) and ICV (Fig. 4c) along with comparison of results from CFD against in vitro. Tracer distribution rate in the caudocranial direction was slower within the LP injection compared to CM and ICV. These three comparative simulations of tracer dispersion indicate that CM and ICV injection facilitate drug delivery to the brain.

The in vitro model supports our assumption for modeling CSF steady streaming as the main driving forces of solute dispersion in slow continuous CSF drug administration. Numerical simulations predicted slightly faster distribution rate compared to in vitro (Fig. 4a1). Overall, the average cranial-directed speed of tracer was ~ 31 cm/h.

**Fig. 5:**
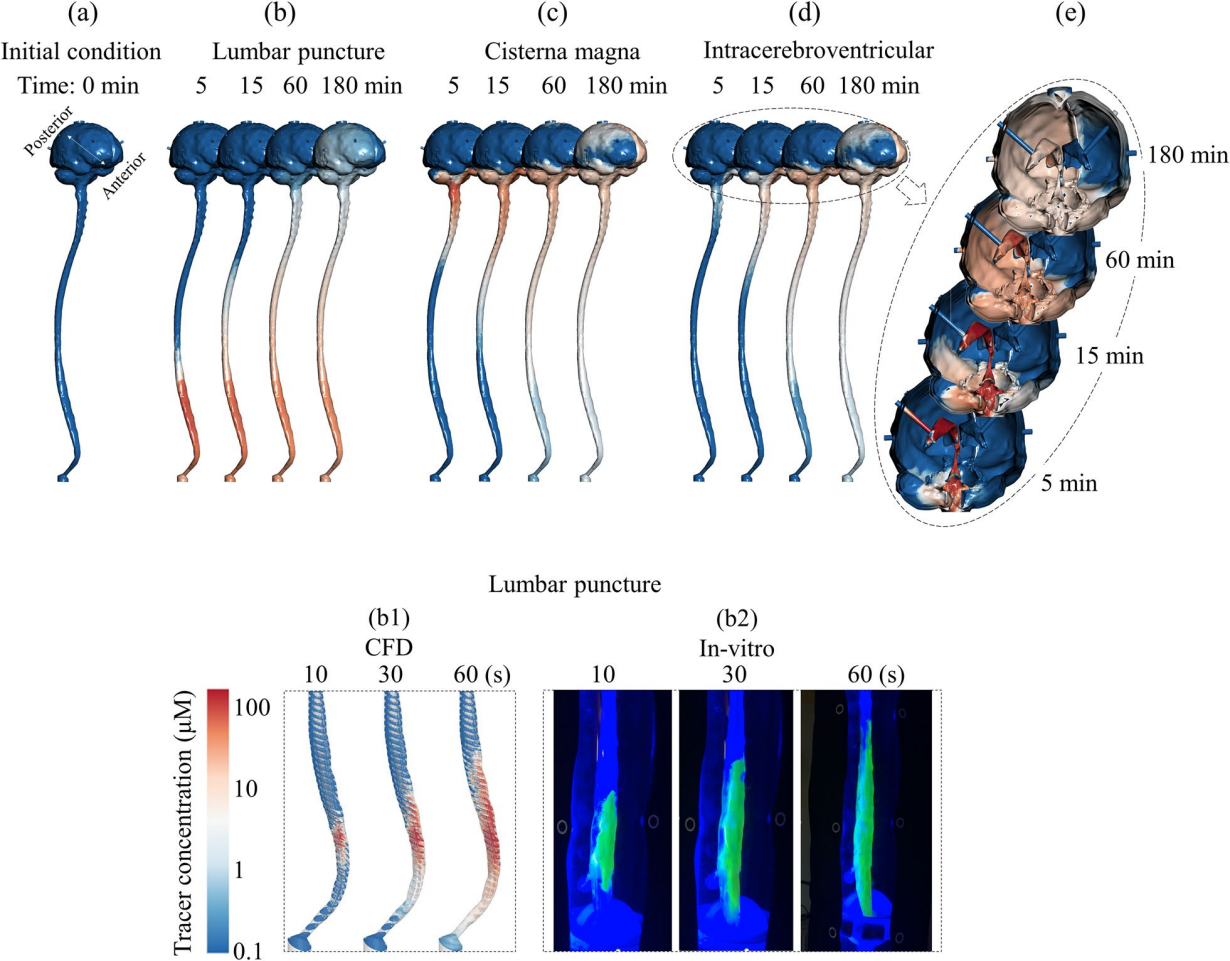
CFD results for 3D tracer concentration profiles versus time for lumbar puncture, cisterna magna and Intracerebroventricular injection. **a** Visualization of tracer concentration before injection starts at time = 0 min. **b**–**d** Visualization of tracer concentration at 5, 15, 60, and 180 min under lumbar puncture, cisterna magna and Intracerebroventricular injection, respectively. **b1**, **b2** Visualization of tracer concentration at 10, 30, and 60 (s) under lumbar puncture injection for CFD simulation and in vitro experiment, respectively. **e** Visualization of tracer concentration at 5, 15, 60, and 180 min inside ventricular system and the cranial SAS for Intracerebroventricular injection

Differences between spatial–temporal cross-sectional average tracer concentration over 3-h obtained from in vitro and CFD were quantified using Bland–Altman plots. A relatively strong linear correlation was observed between the numerical and in vitro results for LP (Fig. 4a3, $R^{2}$ = 0.89, slope = 0.98). Linear correlation for the CM and ICV injection was moderate (Fig. 5b3, c3), $R^{2}$ = 0.70 and 0.58, slope = 0.77 and 0.79). The second set of Bland–Altman plots showed that a greater discrepancy between in vitro and CFD results tended to occur for z-positions closer to the foramen magnum where there is a solid flange to connect the cranium to the spinal SAS for the in vitro model. The 95% confidence intervals for LP, CM and ICV injection were ± 1.20 μM, (± 1.41% of dynamic range), ± 1.15 μM (± 1.35% of dynamic range), ± 0.85 μM (± 1.0% of dynamic range), respectively (Fig. 5a4, b4 and c4).

Figure 5 visualizes tracer distribution contours in the SAS for each injection location at different time points: 5, 15, 60, 180 min. Tracer concentration is also visualized on the ventricular system for the ICV injection (Fig. 5e). A detailed comparison of tracer movement between CFD and in vitro model during the injection and near the tip of the needle was shown in Figs. 5b1, 6b2. In both cases, tracer moved cranially on the anterior side while moving caudally on the lateral side. Overall, the numerical simulations demonstrated needle position drastically impact tracer distribution in the CSF system. These results allow the important conclusion that the needle location critically impact the dispersion speed of CSF administered drugs.

### Effects of physiological changes and tracer injection parameters on tracer exposure

**Fig. 6.**
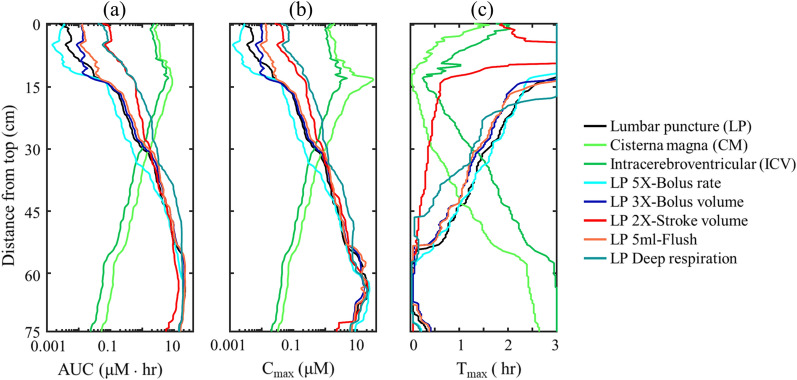
Impact of injection and physiological parameters on tracer concentration over 3-h for numerical simulation result. Results are presented as **a** Area under the curve (AUC), **b** Maximum concentration (C_max_) and **c** Time to the maximum concentration (T_max_)

We calculated the tracer AUC for each axial location along the neuro-axis within the SAS (Fig. 6). In particular, Fig. 6 quantitatively visualizes the impact of different injection and physiological parameters on AUC at different sections along the SAS neuroaxis after 3 h. The effect of the injection parameters on the tracer spatial temporal distributions are not shown since the variation in the plots was not noticeable. A linear decay was observed on both side of the injection location (Fig. 6a). A similar trend was observed for C_max_ (Fig. 6b). Near the injection location, T_max_ occurred when the tracer first appeared at each section (Fig. 6c). Figure 6a shows the AUC after 3-h was ~ 2 log greater with CM and ICV injection. Much greater tracer exposure was achieved in the cranium with CM and ICV injection (3.50 and 2.71 μM · h) compared to average of 0.0026 μM · h for LP (Fig. 6a). On the other hand, C_max_ for the LP was 100X greater (9.74 μM · h) within the spinal SAS compared to average of 0.11 and 0.05 μM · hr for CM and ICV, respectively (Fig. 6b). Maximum tracer concentration occurs near the tip of the needle right after the end of injection for all cases (Fig. 6c).

In light of the noticeable differences brought by the injection location on the tracer distribution, tracer exposure to the spinal SAS was similar to LP for all other simulations indicating that the modifications applied in this study to injection parameters had relatively little impact on the cranial motion of IT delivered drugs due to the short injection time (1–3 min) compared to 3-h simulated post injection (Fig. 6a). However, AUC was almost one order of magnitude greater with stroke volume and deep respiration in the cranial SAS compared to LP. Higher injection rate locally increased C_max_ near the needle tip. However, this effect was negligible as the C_max_ near the needle tip is similar in all cases (Fig. 6b).

**Fig. 7:**
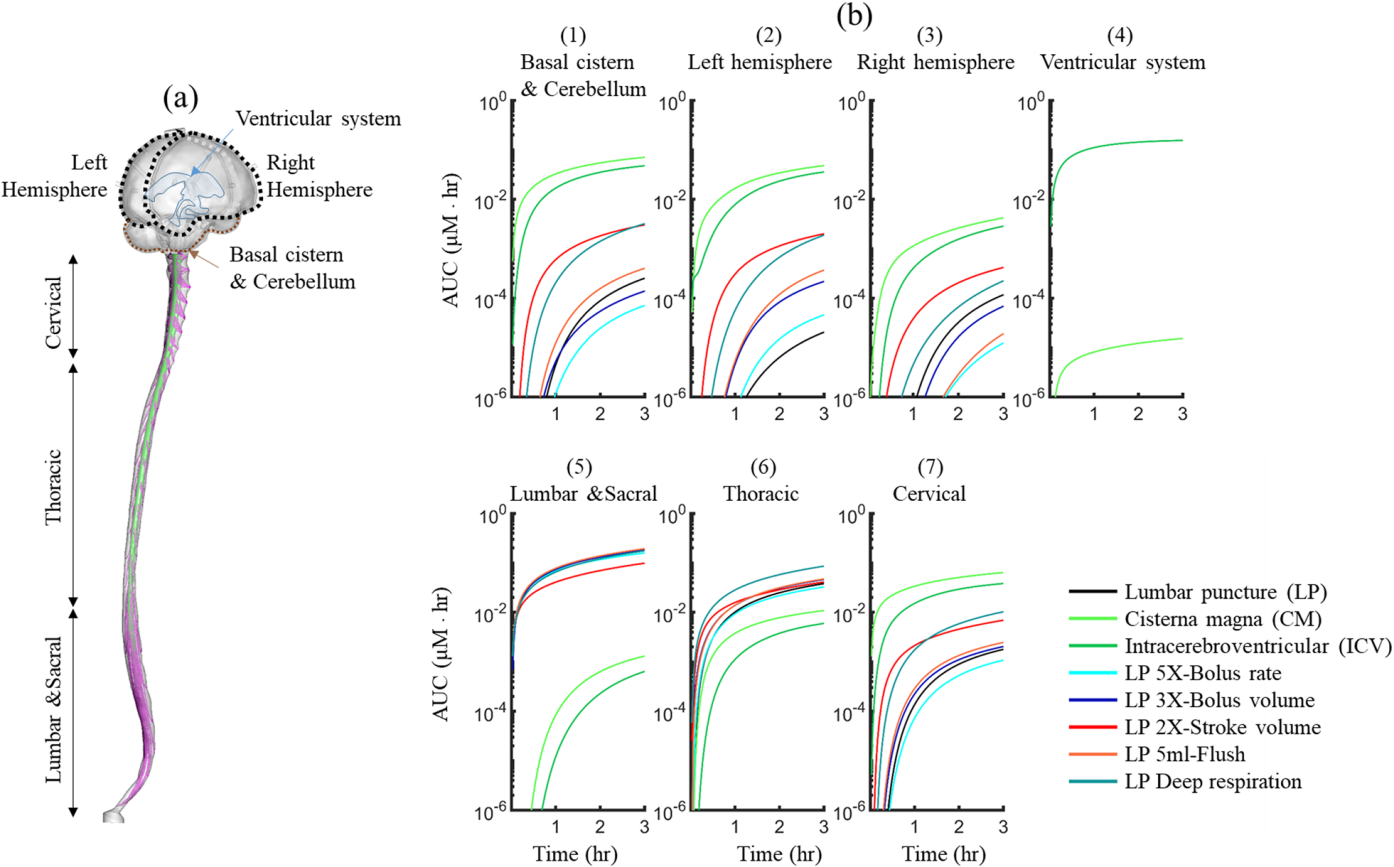
Impact of injection and physiological parameters on tracer concentration over 3-h at each region of the CSF system. Area under the curve (AUC) was calculated as the integration of area under the average concentration at each region. See Fig. 1 for more details about each region in the cranial space

Based on our results, total AUC in the basal cistern and cerebellum was greater than left and right hemisphere for all simulations (Fig. 7b1-3). Higher stroke volume and applying deep respiration led to 10X higher AUC in the cerebellum and basal cistern compared to LP (Fig. 7b1). AUC in the ventricular system was negligible for all simulations except ICV, meaning there was little tracer exposure to the ventricular system (Fig. 7b4). In the spinal region, LP injection with different injection and physiological parameters resulted in a higher AUC compared to CM in ICV injection (Fig. 7b5-7). Different injection parameters showed very small changes on tracer exposure within the lumbar region (Fig. 7b5). Changes in AUC were more noticeable in the cervical region. CM injection showed the greatest tracer exposure in the cervical SAS with ~ 2 log greater AUC compared to LP (Fig. 7b7).

## Discussion

Intrathecal drug administration has gained a significant role in pain management and drug delivery for CNS disease therapeutics. Despite this role, there are few in silico or in vitro trial platforms to study and optimize injection guidelines. A fluid-physics based tool to assist clinicians in choosing the parameters of bolus injection or drug pump settings to administer specific drug doses to the spine or the brain could improve treatment schedules.

This study applied CFD to assess intrathecal drug delivery in an anatomically idealized model of the CSF system with in vitro verification. Overall, our findings suggest that intrathecal drug delivery is sensitive to many factors and that fluid-physics based computational modeling can offer insight into how individual factors may be tuned to produce a desired drug delivery profile. Our approach was to model the tracer dispersion within the flow field using multiphase model and parametrically assess the impact of the following parameters on the tracer spread over 3-h: (a) 5X-Bolus rate increase, (b) 3X-Bolus volume increase, (c) injection location comparing LP, CM and ICV (Table 1).

### Verification of numerical modeling approach

Studies show that wide variability exists in CFD modeling techniques, and the choice of numerical solvers and settings, are complex and can yield disparate results for biofluids simulations [34]. At present, only contrast-enhanced medical imaging methods, direct in vivo fluid or tissue sampling and simplified in vitro systems have been available as tools to quantify drug delivery protocols in the CSF. While these tools are important as verification methods, they are highly invasive, uncontrolled, lack spatial–temporal details, and/or significantly reductionistic. These factors make optimization of CSF drug delivery protocols difficult. As ground truth validation against in vivo measurements is not yet possible, we built an analogous in vitro model to help verify computational results.

Strong correlation of the CFD and corresponding in vitro simulation results were present, indicating robustness of the CFD model to capture the relevant multiphase fluid transport physics. Comparison of spatial–temporal cross-sectional average tracer concentration profiles revealed similar dispersion trends for both CFD and in vitro results under different injection location simulations (Fig. 4). A strong linear correlation was found between CFD and in vitro results under LP (R^2^ = 0.89, Fig. 4a3), and a moderate linear correlation for CM and ICV (R^2^ = 0.6–0.7, Figs. 4b3, 5c3). Bland–Altman plots indicated that 95% of the CFD tracer concentration results were within ~ 2% of the in vitro findings with a mean difference of ~ 0.1% in all three cases (Figs. 4a4, 5b4 and c4). In addition, we conducted a detailed numerical verification uncertainty study (Additional file 1: Appendix) to help further confirm model results. This was based on the ASME standard for verification and validation in CFD with experimental results using the in vitro model [35].

### Injection location alters axial tracer distribution

For the simulations analyzed, injection location was found to alter axial tracer distribution and regional AUC, C_max_, and T_max_ values to the greatest degree (Fig. 6a-c). Taken together, these findings indicate a strong potential for delivery protocols to be optimized to reach a target region(s) of the spine and/or brain with a specific therapeutic dose. In specific, both CM and ICV dramatically increased AUC to brain regions by ~ 2 logs compared to all other simulations and also substantially decreased T_max_ and increased C_max_ values around the brain. However, the CM injection resulted in ~ 2X greater AUC in the left and right hemisphere cortical SAS compared to ICV (Fig. 7b2-3), and it also improved tracer distribution to the basal cistern and cerebellum (Fig. 7b1). In contrast, ICV and CM injections resulted in ~ 2 logs lower AUC values in the lumbar and sacral spine (Fig. 7b5), but approximately 1 log greater AUC in the cervical spine (Fig. 7bC). While CM and ICV injections showed strong similarity in tracer spread around the brain, the overall magnitude of tracer concentration and corresponding AUC around the spinal SAS and brain was lower for ICV since, after 3-h of simulation, ~ 3% of the injected dose remained inside the left lateral ventricle where the tracer was first injected.

Only the ICV and CM injections reached the ventricles (Fig. 4b, c). However, the CM injection, had ~ 4 logs lower AUC in the ventricles (4th ventricle penetration only) compared to ICV; a miniscule value that would likely lack therapeutic benefit. For ICV, tracer did not spread to the contralateral ventricle via connectivity through the intraventricular foramen (Fig. 5e). This is due to a CSF production rate, arising from each ventricle at specified rate of 0.4 mL/min based on average values reported in the literature [32] that was sufficient to limit tracer movement to the contralateral ventricle. From the left lateral ventricle, the tracer moved through the third ventricle, aqueduct of Sylvius and exited the 4th ventricle via the foramen Magendie and Lushka to the CM. If CSF production rate was specified to be smaller, it is likely that the tracer would have been able to move to the contralateral ventricle through oscillatory mixing. This factor could be studied in the future to better understand the potential role, if any, on solute transport, including signaling or inflammatory molecule movement, within the ventricular cisterns. This molecule movement could be altered with CSF production rate, medications, outflow pathway modification such as endoscopic third ventriculostomy, or cerebroventricular shunt placement.

## Steady-streaming CSF velocities impacted solute transport

Comparison of LP versus CM and ICV injections indicates a strong connection of solute transport and geometrically induced steady-streaming flow patterns that occur at different vertebral levels due to the shape of nerve roots, changes in subarachnoid space cross-sectional area, and spinal curvature. As previously quantified [31], steady-streaming in the cervical spine is up to three-times greater than the lumbar spine. Thus, for a CM or ICV injection, tracer distributes symmetrically faster in both the caudal and cranial directions near the injection site compared to LP (Fig. 4). For an injection at the L2-3 level, the tracer spreads slowly towards the head and decelerates locally at T5-6 (~ 35 cm below the foramen magnum), corresponding to a reduction in steady-streaming velocities at that location as previously observed by our research group [31]. For the CM injection, initial peak tracer concentration near the injection site decreased in the axial direction more rapidly due to elevated steady-streaming in the cervical spine (Figs. 5b and 6b C_max_ decrease around injection site). In contrast, for the LP injection, a high concentration tracer region was present for a period ~ 5 min after injection in both CFD (Fig. 5a) and in vitro results (Fig. 4).

### Importance of injection protocol

Parametric modification of injection protocols was found to have impact on tracer distribution; albeit to a smaller degree than injection location. For discussion, we consider the LP case with a 5 mL bolus injected conducted over 1 min to be a “baseline” case for comparison. A 3X increase in bolus volume and addition of a 5 mL flush both increased intracranial AUC to the brain up to 2X compared to baseline (Fig. 6a). In contrast, a 5X increase in bolus rate (25 mL/min) did not improve tracer exposure to the brain in CFD or in vitro results. The reason for the lack of improved tracer movement to the brain under 5X bolus rate may be due to that the steady-streaming velocity introduced by the injection has a shorter duration when the bolus rate was slower at baseline. Although the differences were notable on AUC values, the magnitude of difference in concentration at any time and location (Fig. 6b) did not exceed 10%. This is a relatively small value that would be difficult to detect without the benefit of using the in silico platform with a high degree of repeatability and precision.

### Importance of physiological variables on tracer spread

Variation of physiological variables, namely cardiac-induced and respiratory-induced CSF stroke volume and frequency, were found to have a stronger impact on tracer transport to the brain compared to a 5X bolus rate, 3X bolus volume, or adding a 5 mL flush. In combination, these results indicate that CSF stroke volume and frequency both play an important role and may potentially have a greater impact than modest changes in LP injection protocols such as bolus rate, volume, and flush. An increase in cardiac and respiratory CSF movement improved tracer spread to the brain, basal cistern, and cerebellum up to ~ 2 logs compared to the baseline LP case (Fig. 6a). However, AUC to the brain was only slightly increased around the brain under 2X stroke volume compared to deep respiration, but that advantage decreased over the 3 h simulation period (Fig. 7b1-3). The reason for increased tracer spread along the neuroaxis under 2X stroke volume and deep respiration is because these conditions facilitated a greater steady steaming velocity field, which advected the tracer more quickly away from the initial LP injection site. The deep respiration-induced CSF stroke volume was ~ 6 mL at a frequency of 0.085 Hz (11.7 s) or one breath for every 13 cardiac cycles (Fig. 2). Cardiac-induced CSF flow oscillations had a stroke volume of 1 mL at 1.11 Hz (0.9 s). The relative importance of the examined physiological changes against injection protocols may help explain the large degree of experimental variability often observed in pre-clinical non-human primate studies where factors like respiratory and cardiac rate may vary across animals under investigation.

### Comparison of results to previous computational models

Our in silico and in vitro trials support previous study findings in terms of tracer transport timing, dynamics, and overall study findings. Table 1 provides a summary of in silico trials conducted in the past and their key findings in terms of how specific physiologic, injection protocols, and injection locations either increased or decreased tracer transport to the brain. Tangen et al. [33] used an anatomically idealized CFD model to study the impact of various parameters such as: injection volume, heart rate, and drug uptake for a period of 1 h after injection. They found that the greatest drug exposure to the cranial region was achieved by increasing bolus volume and found that addition of a 10 mL flush increased rostral transport of the tracer up to 2X compared to a 5 mL flush. In our study, addition of a 5 mL flush had a similar increase in brain biodistribution compared to baseline ranging from 3 to 5X (Fig. 6a). Kuttler et al. used a steady-streaming-based CFD technique to simulate drug dispersion within the spinal SAS and showed arrival of an LP injected drug 10 cm below the foramen magnum after 60 min of simulation time. In our study, a slightly slower transport timing along the spine was observed, with initial tracer arrival 10 cm below the foramen magnum (25 cm in our model) occurring at ~ 60 to 90 min (Fig. 4a2) and T_max_ occurring from ~ 75 to 105 min post-LP injection (Fig. 7c). Similar to Haga et al. [36], tracer in the cervical spine was found to move cranially along the ventral and dorsal SAS and caudally in the lateral SAS (Fig. 5b1, b2). Haga et al., also previously showed that moving the injection location closer to the brain by one vertebral level increased tracer concentration in the cranial direction [36].

### Comparison of results to in vivo studies in the literature

A number of studies involving intrathecal drug injection based on MR imaging have been conducted in monkeys [37] and humans [38, 39] (Table 1). Overall, our tracer dispersion results show a similar trend in tracer arrival time to the cranial base post LP injection as observed in vivo. Watts et al. observed that LP gadolinium injection resulted in very little tracer movement to the ventricular cisterns of the brain. Our LP simulations had a similar result. These findings verify that contrast entering the ventricular system, if any, is rapidly diluted by CSF production outflow or that CSF production does not allow tracer in the cortical subarachnoid space to move up stream into the ventricular space [38].

Tracer concentration in the cranial region displayed similar features in terms of timing and distribution to Watts et al. around the brain with greater concentrations at the basal cistern and cerebellum compared to cortical subarachnoid space [38]. Tangen et al. showed tracer distribution along the neuroaxis of three non-human primates over a period of two hours using in vivo MRI and PET imaging. They found that the tracer reached the cervical region of the spine after approximately two hours. Like their study, our result showed that cerebral targets are reachable with LP injection within two hours (Figs. 4a, 5b), albeit our simulation was conducted for a human subject and did not include model validation with in vivo data collected on humans. Verma et al. used PET imaging to quantify LP injection neuraxial spread in healthy humans [8]. Using an injection protocol comparable to the LP case in our study, they found that tracer reached the cranial base after approximately 3-h (Fig. 5b), a time slightly slower than observed in our study. Verma et al. also showed that an increase in bolus volume increased the drug concentration around the brain after 1-h which is in agreement with our results for a 3X-Bolus volume injection (Fig. 7b1-3).

Eide et al. applied contrast enhanced MRI using gadolinium as a CSF tracer to investigate lymphatic drainage of CSF to cervical lymph nodes [9]. In their study, an injection of 0.5 mL of 1.0 mmol/mL gadolinium was injected into the lumbar subarachnoid space. At 2–4 h post injection, tracer reached the thalamus, inferior frontal gyrus and pons with a similar degree of change (about 8%) in signal unit ratio. In our study, we found similar results for the LP simulation. Figure 4b shows a 7–9% increase in concentration within the cortical CSF region after 3-h. It should be noted that because the gadolinium signal is non-linear with concentration, exact comparison of results is not possible.

A study by Wolf et al. showed that CM injection of two different contrast agents (10 μl of ^111^In-DTPA/^99m^Tc-sestamibi) filled the basal intracranial cisterns and cerebellum within the first 15 min [40]. Similar to our study, CM injection showed that, after 15 min, tracer reached the cerebellum (Fig. 5c). Another study by Meyer et al. [41], showed significant improvement of delivery to the brain using CM and ICV injection (~ 10X less exposure in the spinal region). Similar to our result, total exposure of the tracer in the spinal region was ~ 10X less compared to LP (Fig. 6A, B).

### Limitations

In order to have the CFD and in vitro model consistent for validation of the results, we used a rigid wall instead of attempting to use dynamic mesh technique for CFD model, previously described by Khani et al. [42]. A future, more complete, model of CSF dynamics could incorporate a dynamic mesh with non-uniform flow and also include a glymphatic system with a term incorporating absorption into the tissue.

Our model did not include Fluid Structure Interaction (FSI) due to challenges, including computational times required for this method and limited computational resources available. This is an important aspect since the physiology includes FSI and it could be addressed in future studies. Other limitations including physiologic interactions related to connected systems should be addressed in the future studies. For example, in vivo cardiac-related CSF dynamics are coupled to periodic volume changes within the cranial cavity subject to the Monro-Kellie doctrine [43]. Also, the systolic increase in arterial blood pressure and volume leads to displacement of other materials including venous blood and CSF.

In the present study of tracer transport, the effect of diffusion was neglected due to the relatively slow rate of molecular diffusion compared to streaming transport [31]. This assumption was verified by comparison of in vitro versus CFD solute spatial–temporal transport (Fig. 4). The present study also did not test injection parameters such as injection angle and/or dorsal ventral injection which were not expected to have a large impact on the relatively long-time scale of 3-h. Although CM administration showed increased brain exposure, the clinical applicability of this approach is more limited compared to LP due to risk and complications than can occur due to CM [44]. Further simulations could study the impact of constant slow-rate infusion via LP instead of bolus administration and find the rate of infusion and or time that may emulate brain "bathing" and quanitify results compared to CM injection.

The present study did not include any in vivo validation of results as this required invasive procedures which were not possible for our research team to collect at the time of the study. Thus, the computational model was verified with controlled in vitro experiments. Future studies are needed for validation of CFD results with real-world imaging in human participants.

Our model did not include tracer absorption due to microvasculature into the CNS tissue. This could have an impact on tracer concentration (particularly for LP) over long time periods. Our approach was focused on early pharmacokinetic transport in the CSF within a relatively short biologic timescale. In addition, the computational geometry did not include arachnoid trabeculae, structures that have been shown in the past to potentially alter the CSF flow field [25]. The proposed modeling framework can be modified to investigate the impact of arachnoid trabeculae in future studies.

Our 3D model had some limitations regarding the anatomy, mainly due to the highly complex nature of the CSF system. The CFD model did not distinguish compliance within the intracranial compartments and spinal cord tissue since the geometry of the spinal canal is extremely complex. This important aspect can be addressed in the future study.

Both CM and ICV injections showed that the tracer spread earlier to the left hemisphere compared to the right hemisphere for all simulations conducted in this study (Fig. 7). The reason for asymmetrical spread within the cranium is likely due to a slight difference in hydraulic resistance across the cortical convexity for the geometry applied in our study. A similar asymmetry in tracer distribution was observed in our previous study [31] that simulated blood clearance from CSF. Future studies could investigate the impact of parametric changes in cortical SAS geometry and other factors such as spinal curvature and spinal cord eccentricity.

## Conclusions

This study addressed intrathecal solute transport within the complete CSF system and the role of injection location (LP, CM and ICV), injection rate and volume, flush, as well as physiologic factors of cardiac-induced and deep respiration-induced CSF stroke volume on tracer distribution. For the injection scenarios analyzed in our study, CM injection was found to be the best way to improve tracer delivery to the brain; albeit CM injection can present significant risk to the patient. Injection location was found to affect the tracer distribution profile more than other injection parameters. LP injection generated a parabolic concentration profile within the spinal SAS. ICV injection enhanced tracer distribution inside the brain while reducing the peak concentration in the cerebellum compared to cervical injection. The effect of injection bolus volume and rate on maximum tracer exposure within the model was at most 2X greater compared to LP while higher stroke volume and deep respiration resulted in ~ 2 logs greater tracer exposure within the cranial SAS. The computational modeling approach provides detailed insight into how the tracer concentration is impacted within the CSF while in vitro modeling generated results faster. The threshold at which injection location would make a clinically significant difference is not yet known. We cannot extract general working guidelines based on these results; however, they suggest choosing an injection location in close proximity to the targeted therapeutic area. Also, that relatively modest modification of the injection protocols can have a noticeable impact for LP injections.

## Supplementary Information

**Additional file 1.** Spatial-temporal experimental (EXP) and computational fluid dynamics (CFD) results. EXP results provided only for verification studies conducted for the ICV, CM, and LP case (Fig. 4). CFD results provided for all injection scenarios. Rows represent axial position along the model with a spacing of 380 microns (76 cm total model length) and top row representing the cranial end of the model. Columns represent time steps with a resolution of 900 ms per step (180 min total simulation time) and left column representing time = 0.

## Data Availability

The data that support the findings of this study are available from the corresponding author, [BAM], upon reasonable request.

## Abbreviations

CM: Cisterna magna
HR: Heart rate
LP: Lumbar puncture
AUC: Area under the curve
CFD: Computational fluid dynamics
CNS: Central nervous system
CSF: Cerebrospinal fluid
ICV: Intracerebroventricular injection
MRI: Magnetic resonance imaging
SAS: Subarachnoid spaces
