# Supplementary material for: Human in silico trials for parametric computational fluid dynamics investigation of cerebrospinal fluid drug delivery: impact of injection location, injection protocol, and physiology
Source: Fluids Barriers CNS. 2022 Jan 28;19:8. doi: 10.1186/s12987-022-00304-4 (PMC8796513; doi:10.1186/s12987-022-00304-4)
Supplement: Supplementary file 1 — Additional file 1. Spatial-temporal experimental (EXP) and computational fluid dynamics (CFD) results. EXP results provided only for verification studies conducted for the ICV, CM, and LP case (Fig. 4). CFD results provided for all injection scenarios. Rows represent axial position along the model with a spacing of 380 microns (76 cm total model length) and top row representing the cranial end of the model. Columns represent time steps with a resolution of 900 ms per step (180 min total simulation time) and left column representing time = 0. [file 12987_2022_304_MOESM1_ESM.docx]

***Additional data***

*Verification uncertainty*

The ASME standard for verification and validation in CFD was used for validating our CFD model by comparing the CFD predictions with experimental results using the in vitro model (35). Validation uncertainty ($U_{v}$) is defined as composition of uncertainty in the numerical simulation ($U_{num}$) and in vitro experiments ($U_{EXP}$) as:

$U_{v}^{2}=U_{num}^{2}+U_{EXP}^{2}$ (1)

$U_{EXP}=\frac{2S_{X}}{\sqrt{N}}$ (2)

(3)

$\bar{X}(z,t)=\frac{\sum_{j=1}^{N} X_{j}(z,t)}{N}$ (4)

Where $S_{X}(z,t)$is the in vitro experiments standard deviation and $\bar{X}(z,t)$ is the in vitro experiments mean value.$X_{j}(z,t)$represents the cross-sectional average tracer concentration at each time and location for each individual in vitro experiment and *N* is the number of experiments. It should be noted that we ignored the systematic standard uncertainty of the measurements after careful calibrations of our instruments. We also ignored the uncertainty of input parameters due to the unknown sensitivity coefficients. Therefore, the validation uncertainty in our study is less conservative than it should be, which adds a stricter validation criterion on our CFD results.

The numerical simulation uncertainty for CSF simulation was previously described by khani et al. using the L2 norm and factor of safety method (45, 46). As summary, $U_{num}$ was defined based on local error of cross-sectional average tracer concentrations on a systematically refined grid triplet:

$U_{FS}=FS(P)\left|\delta_{RE}\right|\left\{\begin{matrix} (2.45-0.85P)\left|\delta_{RE}\right| 0<P\leq1 \\ 16.4P-14.8\left|\delta_{RE}\right| P>1 \end{matrix}\right.$ (5)

Where *P* is the ratio of the estimated order of accuracy to the theoretical order of accuracy and $\delta_{RE}$ is the estimated numerical error using Richardson extrapolation.

The factor of safety method gives additional confidence in the tracer distribution results. Uncertainties for all cases was below one for most regions except near the injection locations where initial transport dynamic details varied locally for a short period of time.
